# Focusing on the Golden Ball Metaheuristic: An Extended Study on a Wider Set of Problems

**DOI:** 10.1155/2014/563259

**Published:** 2014-08-03

**Authors:** E. Osaba, F. Diaz, R. Carballedo, E. Onieva, A. Perallos

**Affiliations:** Deusto Institute of Technology (DeustoTech), University of Deusto, Avenida Universidades 24, 48007 Bilbao, Spain

## Abstract

Nowadays, the development of new metaheuristics for solving optimization problems is a topic of interest in the scientific community. In the literature, a large number of techniques of this kind can be found. Anyway, there are many recently proposed techniques, such as the artificial bee colony and imperialist competitive algorithm. This paper is focused on one recently published technique, the one called Golden Ball (GB). The GB is a multiple-population metaheuristic based on soccer concepts. Although it was designed to solve combinatorial optimization problems, until now, it has only been tested with two simple routing problems: the traveling salesman problem and the capacitated vehicle routing problem. In this paper, the GB is applied to four different combinatorial optimization problems. Two of them are routing problems, which are more complex than the previously used ones: the asymmetric traveling salesman problem and the vehicle routing problem with backhauls. Additionally, one constraint satisfaction problem (the n-queen problem) and one combinatorial design problem (the one-dimensional bin packing problem) have also been used. The outcomes obtained by GB are compared with the ones got by two different genetic algorithms and two distributed genetic algorithms. Additionally, two statistical tests are conducted to compare these results.

## 1. Introduction

Today, optimization problems receive much attention in artificial intelligence. There are several types of optimization, such as numerical [1], linear [2], continuous [3], or combinatorial optimization [4]. Typically, problems arising in these fields are of high complexity. Additionally, many of the problems arising in optimization are applicable to the real world. For these reasons, in the literature, many different techniques designed to be applied to these problems can be found.

Some classical examples of these techniques are the simulated annealing [5], the tabu search [6], the genetic algorithm (GA) [7, 8], ant colony optimization [9], or the particle swarm optimization [10]. Since their proposal, all these metaheuristics have been widely applied in a large amount of fields. In fact, these techniques are the focus of many research studies nowadays [11–14].

Despite the existence of these conventional algorithms, the development of new metaheuristics for solving optimization problems is a topic of interest in the scientific community. On the one hand, optimization problems represent a great challenge because they are hard to solve. For this reason, the development of new techniques that outperform the existing ones is a topic of interest for the researchers. On the other hand, optimization problems (such as routing problems) are very important from a social perspective. This is because their resolution directly affects the economy and sustainability in terms of cost reduction and energy saving.

In this way, there have been many recently proposed metaheuristics, which have been successfully applied to various fields and problems. One example is the imperialist competitive algorithm (ICA) [15]. This population based metaheuristic, proposed by Gargari and Lucas in 2007, is based on the concept of imperialisms. In ICA, individuals are called countries and they are divided into various empires, which evolve independently. Throughout the execution, different empires battle each other with the aim of conquering their colonies. When one empire conquers all the colonies, the algorithm converges into the final solution. Some examples of its application can be seen in recent papers [16, 17]. Another example is the artificial bee colony. This technique was proposed in 2005 by Karaboga [18, 19] for multimodal and multidimensional numeric problems. Since then, it has been used frequently in the literature for solving different kinds of problems [20–22]. The artificial bee colony is a swarm based technique which emulates the foraging behaviour of honey bees. The population of this metaheuristic consists in a colony, with three kinds of bees: employed, onlooker, and scout bees, each with a different behaviour. The harmony search, presented by Geem et al. in 2001, is another example [23, 24]. This metaheuristic mimics the improvisation of music players. In this case, each musical instrument corresponds to a decision variable; a musical note is the value of a variable; and the harmony represents a solution vector. With the intention of imitating the musicians in a jam session, variables have random values or previously memorized good values in order to find the optimal solution. This algorithm is also used frequently in the literature [25–27].

Bat-inspired algorithm is a more recent technique [28, 29]. This metaheuristic, proposed by Yang in 2010, is based on the echolocation behaviour of microbats, which can find their prey and discriminate different kinds of insects even in complete darkness. Yang and Deb proposed the cuckoo search algorithm in 2009 [30, 31]. On this occasion, as authors claim in [30], this metaheuristic is based on the obligate brood parasitic behaviour of some cuckoo species in combination with the Levy flight behaviour of some birds and fruit flies. Another recently developed technique which is very popular today is the firefly algorithm [32, 33]. This nature-inspired algorithm is based on the flashing behaviour of fireflies, which act as a signal system to attract other fireflies. Like the aforementioned techniques, these metaheuristics have been the focus of several research [34–40] and review papers [41–43].

As can be seen, there are many metaheuristics in the literature to solve optimization problems. Although several techniques have been mentioned, many other recently developed ones could be cited, such as the spider monkey optimization [44] or seeker optimization algorithm [45]. This large amount of existing techniques demonstrates the growing interest in this field, on which several books, special issues in journals, and conferences proceedings are published annually. Moreover, combinatorial optimization is a widely studied field in artificial intelligence nowadays. Being NP-Hard [46], a lot of problems arising in this field are particularly interesting for the researchers. This kind of optimization is the subject of a large number of works every year [47–49]. This scientific interest is the reason why this study is focused on this sort of optimization.

This paper is focused on one recently proposed metaheuristic called Golden Ball (GB). This technique is a multiple-population based metaheuristic, and it is based on soccer concepts. A preliminary version of the GB and some basic results were firstly introduced in 2013 by Osaba et al. [50]. Furthermore, the final version of the GB and its practical use for solving complex problems have been presented this very year (2014) by the same authors [51]. In that paper, the GB is introduced, and it is compared with some similar metaheuristics of the literature. In addition, it is successfully applied to two different routing problems: the traveling salesman problem (TSP) [52] and the capacitated vehicle routing problem (CVRP) [53]. Additionally, the results obtained by GB were compared with the ones obtained by two different GAs and two distributed genetic algorithms (DGA) [54, 55]. As a conclusion of that study, it can be said that the GB outperforms these four algorithms when it is applied to the TSP and CVRP.

The authors of that study claim that GB is a technique to solve combinatorial optimization problems. Even so, they only prove its success with two simple routing problems, the TSP and the CVRP. This is the reason that motivates the work presented in this paper. Thus, the objective of this paper is to verify if the GB is a promising metaheuristic to solve combinatorial optimization problems, performing a more comprehensive and rigorous experimentation than that presented to date. Thereby, in this research study, the GB is applied to four different combinatorial optimization problems. Two of them are routing problems, which are more complex than the ones used in [51]: the asymmetric traveling salesman problem (ATSP) [56] and the vehicle routing problem with backhauls (VRPB) [57]. Furthermore, in order to verify that the GB is also applicable to other types of problems apart from the routing ones, two additional problems have also been used in the experimentation, the n-queen problem (NQP) [58] and the one-dimensional bin packing problem (BPP) [59]. As in [51], the results obtained by GB are compared with the ones obtained by two different GAs and two DGAs. Besides, with the objective of performing a rigorous comparison, two statistical tests are conducted to compare these outcomes: the well-known normal distribution *z*-test and the Friedman test.

The rest of the paper is structured as follows. In Section 2, the GB is introduced. In Section 3, the problems used in the experimentation are described. Then, in Section 4, the experimentation conducted is described. In Section 5, the results obtained are shown and the statistical tests are performed. This work finishes with the conclusions and future work (Section 6).

## 2. Golden Ball Metaheuristic

**Figure 1:**
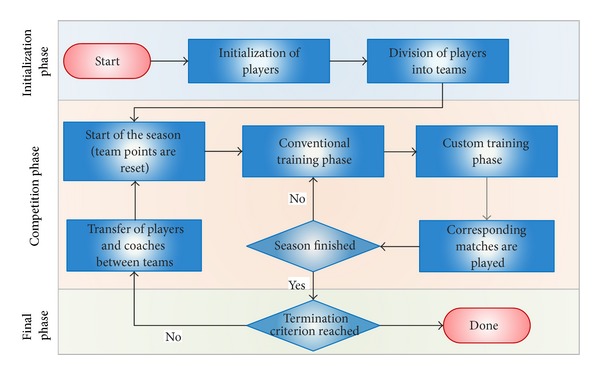
Flowchart of GB metaheuristic.

In this section, the GB is described. As has been mentioned in Section 1, the GB is a multiple-population based metaheuristic which takes several concepts related to soccer. To begin with, the technique starts with the initialization phase (Section 2.1). In this first phase, the whole population of solutions (called players) is created. Then, these players are divided among the different subpopulations (called teams). Each team has its own training method (or coach). Once this initial phase has been completed, the competition phase begins (Section 2.2). This second phase is divided in seasons. Each season is composed of weeks, in which the teams train independently and face each other creating a league competition. At the end of every season, a transfer procedure happens, in which the players and coaches can switch teams. The competition phase is repeated until the termination criterion is met (Section 2.3). The entire procedure of the technique can be seen in Figure 1. Now, the different steps that form the proposed technique are explained in detail.

### 2.1. Initialization Phase

As has been said, the first step of the execution is the creation of the initial population *P*. The initial population is composed of *PT* · *TN* number of solutions *p*_*i*_, called *players*. Note that *PT* is the number of players per team, and *TN* is the number of teams. Additionally, both parameters must have a value higher than 1.

After the whole population *P* is created, all the *p*_*i*_ are randomly divided in the *TN* different teams *t*_*i*_. Once the players are divided between the different teams, they are represented by the variable *p*_*ij*_, which means* the player number j of the team i*. The total set of teams *T* forms the league. All these concepts may be represented mathematically as follows:

(1) P:{p1,p2,p3,p4,p5,…,pPT∗TN}T:{t1,t2,t3,t4,…,tTN}Team t1:{p11,p12,p13,…,p1PT}Team t2:{p21,p22,p23,…,p2PT}⋮Team tTN:{pTN1,pTN2,…,pTN PT}PT=Number  of  players  per  team,TN=Total  number  of  teams  of  the  system.

Furthermore, every *p*_*ij*_ has its own quality, which is represented by the variable *q*_*ij*_ (quality of the player *i* of team *j*). This variable is represented by a real number, which is determined by a cost function *f*(*p*_*ij*_). This function depends on the problem. For example, for some routing problems, this function is equivalent to the traveled distance. On the other hand, for the NQP, for instance, this function is the number of collisions. In addition, each *t*_*i*_ has a captain (*p*_*i*cap_), which is the player with the best *q*_*ij*_ of their team. To state this in a formal way, consider

(2) $$\begin{array}{c} p_{i\text{cap}}=p_{ik}\in t_{i}⟺\forall j\in \left\{1,\ldots ,PT\right\}:q_{ik}\geq q_{ij}. \end{array}$$

It should be borne in mind that, depending on the problem characteristics, the objective is to minimize or maximize *f*(*p*_*ij*_). In the problems used in this paper, for example, the lower the *q*_*ij*_ is, the better the player is.

Moreover, each team has a strength value associated with *TQ*_*i*_. This value is crucial for the matches between teams (Section 2.2.2). It is logical to think that the better the players are, the stronger a team is. Thereby, if one team is strong, it can win more matches and it can be better positioned in the classification of the league. In this way, the strength value of a team *t*_*i*_ is equal to the average of the *q*_*ij*_ of the players of that team. *TQ*_*i*_ can be expressed by the following formula:

(3) $$\begin{array}{c} TQ_{i}=\frac{\sum_{j=1}^{PT}q_{ij}}{PT}. \end{array}$$

Once the initialization phase is completed, the competition phase begins. This phase is repeated iteratively until the ending criterion is met.

### 2.2. Competition Phase

This is the central phase of the metaheuristic. In this stage, each team evolves independently and improves its *TQ*_*i*_ (Section 2.2.1). Additionally, in this phase, the teams face each other, creating a league competition (Section 2.2.2). This league helps to decide the player transfers between teams (Section 2.2.3). The competition stage is divided into seasons (*S*_*i*_). Each *S*_*i*_ has two different periods of player transfers. In each season, every team face each other twice. In this way, each team plays 2NT-2 matches in a season. Lastly, an amount of training sessions equal to the number of matches played is performed.

#### 2.2.1. Training of Players

As in real life, trainings are the processes that make players improve their quality. In GB, each *t*_*i*_ has its own training method, and some of them are more effective than others. This fact makes some teams improve more than others. There are two kinds of training methods in GB:* conventional trainings* and* custom trainings*.

*Conventional trainings* are those that are performed regularly throughout the season. This type of training is applied individually for each player. A training method is a successor function, which works on a particular neighborhood structure in the solution space. Taking the TSP as example, one training function could be the 2-opt [60]. As has been said, each team has its own training function, which acts as the coach of the team. The training function is assigned randomly at the initialization phase. Thereby, each *p*_*ij*_ uses the method of its team. For each training session, the function is applied a certain number of times, and the *p*_*ij*_′ generated is accepted if *q*_*ij*_′ > *q*_*ij*_. Besides, this process could make a change in the *p*_*i*cap_ of a team, if one *p*_*ij*_ outperforms the quality of its captain.

**Figure 2:**
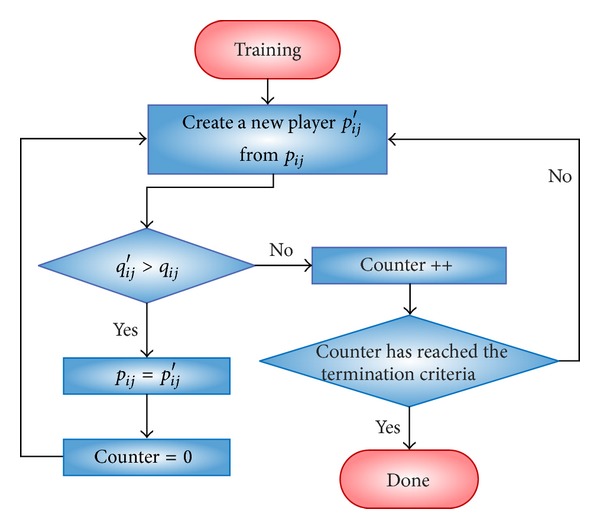
Workflow of a training process.

It is worth mentioning that one training session has its own termination criterion. A training session ends when there is a number of successors without improvement in the quality of the *p*_*ij*_ trained. This number is proportional to the neighborhood of the team training function. For example, taking the 2-opt and a 30-noded TSP instance, the training ends when there are *n*/4 + ∑_*k*=1_^*n*/4^ *k* (the size of its neighborhood) successors without improvement, with *n* being the size of the problem (30).  Figure 2 schematizes this process.

Furthermore, the fact that every *t*_*i*_ explores the space solution in a different way increases the exploration and exploitation capacity of the GB. This fact occurs because of the use of a different training method for each team. Besides, this is enhanced by the fact that players can change their teams.

On the other hand, the procedure of* custom trainings* is different. These trainings are performed when one *p*_*ij*_ receives a number of conventional training sessions without experiencing any improvement (in this study, this number has been set in *PT*/2). When this fact happens, it can be considered that *p*_*ij*_ is trapped in a local optimum. A* custom training* is conducted by two players: the trapped *p*_*ij*_ and the *p*_*i*cap_ of their team. The purpose of these operations is to help *p*_*ij*_ to escape from the local optimum and to redirect them to another promising region of the solution space. From a practical point of view, custom training combines two players (like the crossover operator of a GA), resulting in a new player *p*_*ij*_′ who replaces *p*_*ij*_. Taking the TSP as example, a function that combines the characteristics of two players could be the order crossover (OX) [61] or the partially mapped crossover [62]. The custom training helps a thorough exploration of the solution space.

#### 2.2.2. Matches between Teams

**Figure 3:**
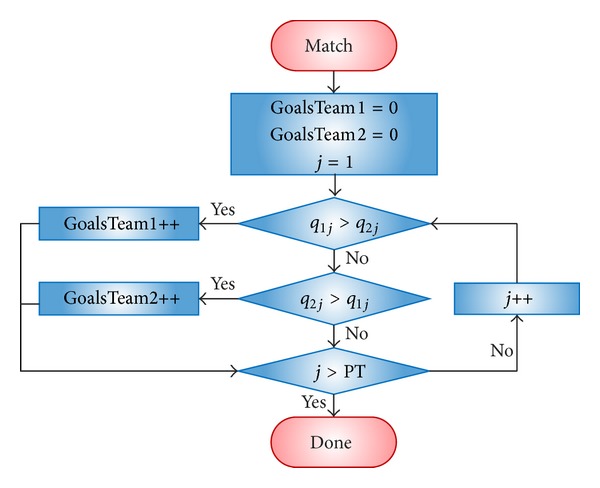
Flowchart of a match.

In GB, as in the real world, two teams (*t*_*j*_, *t*_*k*_) are involved in a match. Each match consists in *PT* goal chances, which are resolved in the following way: first, the players of both teams are sorted by quality (in descending order). Then, each *p*_*ij*_ faces *p*_*ik*_. The player with the highest quality wins the goal chance, and their team scores a goal. As can be surmised, the team that achieves more goals wins the match. Furthermore, the team that wins the match obtains 3 points and the loser obtains 0 points. If both teams obtain the same number of goals, each one receives one point. These points are used to perform a team classification, sorting the teams by the points obtained in a whole season.  Figure 3 shows the flowchart of a match.

#### 2.2.3. Player Transfers between Teams

The transfers are the processes in which the teams exchange their players. There are two types of transfers in GB:* season transfers* and* special transfers*. The former are the conventional ones, and they take place twice in a *S*_*i*_. In these transfers, the points obtained by each team and its position in the league are crucial factors. In this way, in the middle and the end of each *S*_*i*_, the teams placed in the top half of the league classification “hire” the best players of the teams located on the lower half. Moreover, teams of the bottom half receive in return the worst players of the top teams. In addition, the better the position of the team is, the better the player it receives is. In other words, the best *t*_*i*_ is reinforced with the best player of the worst team of the league. Furthermore, the second *t*_*i*_ obtains the second best *p*_*ij*_ of the penultimate team, and so on. Finally, if the league has an odd number of teams, the team placed in the middle position does not exchange any *p*_*ij*_.

On the other hand, the* special transfers* are sporadic exchanges that can occur at any time during a season. If one player receives a certain number of conventional and custom trainings without experiencing any improvement, they changes their team (in this study, this number has been set in PT conventional training sessions without improvement). This change is made in order to obtain a different kind of training. Besides, it is no matter whether the destination team has less *TQ*_*i*_ than its current team. Additionally, with the aim of maintaining the *PT* per team, there is an exchange with a random player of the destination team.

As authors said in [51], these interchanges help the search process. This neighborhood changing improves the exploration capacity of the technique.

Lastly, it is noteworthy that another sort of transfer exists in GB. In this case, these transfers are not performed with players, but with team coaches. This process has been called* cessation of coaches*. In each period of* season tranfers*, the teams positioned in the bottom half of the league classification change their training form. This change is made hoping to get another kind of training which improves the performance and the *TQ*_*i*_ of the team. This training exchange is performed randomly among all the training types existing in the system, allowing repetitions between different *t*_*i*_. This random neighborhood change increases the exploration capacity of the metaheuristic.

### 2.3. Termination Criterion

The termination criterion is a critical factor in the development of a metaheuristic. It must be taken into account that this criterion has to allow the search to examine a wide area of the solution space. On the other hand, if it is not strict enough, it can lead to a considerable waste of time. In this way, the termination criterion of the GB is composed of three clauses:

(4) $$\begin{array}{c} \overset{TN}{\underset{i=1}{\sum }}q_{i\text{cap}}^{\prime }\leq \overset{TN}{\underset{i=1}{\sum }}q_{i\text{cap}},\\ \overset{TN}{\underset{i=1}{\sum }}{TQ}_{i}^{\prime }\leq \overset{TN}{\underset{i=1}{\sum }}TQ_{i},\\ {BestSol}^{\prime }\leq BestSol. \end{array}$$

In other words, the execution of the GB finishes when (1) the sum of the quality *q*_*i*cap_′ of all the captains is not improved over the previous season, (2) the sum of the strengths *TQ*_*i*_′ of all the teams has not been improved compared to the previous season, and (3) there is no improvement in the best solution found (*BestSol*′) in relation to the previous season. When these three conditions are fulfilled, the *p*_*ij*_ with the best *q*_*ij*_ of the system is returned as the final solution.

## 3. Description of the Used Problems

As has been mentioned in the introduction of this study, four combinatorial optimization problems have been used in the experimentation conducted. In this section, these problems are described. The first two are routing problems: the ATSP (Section 3.1) and the VRPB (Section 3.2). Besides, with the aim of verifying whether the GB is also a promising technique with other kinds of problems apart from the routing ones, the NQP (Section 3.3) and the BPP (Section 3.4) have been used.

It is important to highlight that the objective of the present paper is not to find an optimal solution to these problems. In fact, in the literature, there are multiple efficient techniques with this objective. Instead, these four problems have been used as benchmarking problems. In this way, the objective of using them is to compare the performance of the GB with the one of the GAs and DGAs and to conclude which obtains better results using the same parameters and functions.

### 3.1. Asymmetric Traveling Salesman Problem

As the symmetric version of this problem (the TSP), the ATSP has a great scientific interest, and it has been used in many research studies since its formulation [63, 64]. This problem can be defined as a complete graph *G* = (*V*, *A*), where *V* = {*v*_1_, *v*_2_,…, *v*_*n*_} is the set of vertexes which represents the nodes of the system and *A* = {(*v*_*i*_, *v*_*j*_) : *v*_*i*_, *v*_*j*_ ∈ *V*, *i* ≠ *j*} is the set of arcs which represents the connection between nodes. Additionally, each arc has an associated distance cost *d*_*ij*_. Unlike in the TSP, in the ATSP, the distance cost between two nodes is different depending on the direction of the flow; that is, *d*_*ij*_ ≠ *d*_*ji*_. Thereby, the objective of the ATSP is to find a route that, starting and finishing at the same node, visits every *v*_*i*_ once and minimizes the total distance traveled. In this way, the objective function is the total distance of the route.

**Figure 4:**
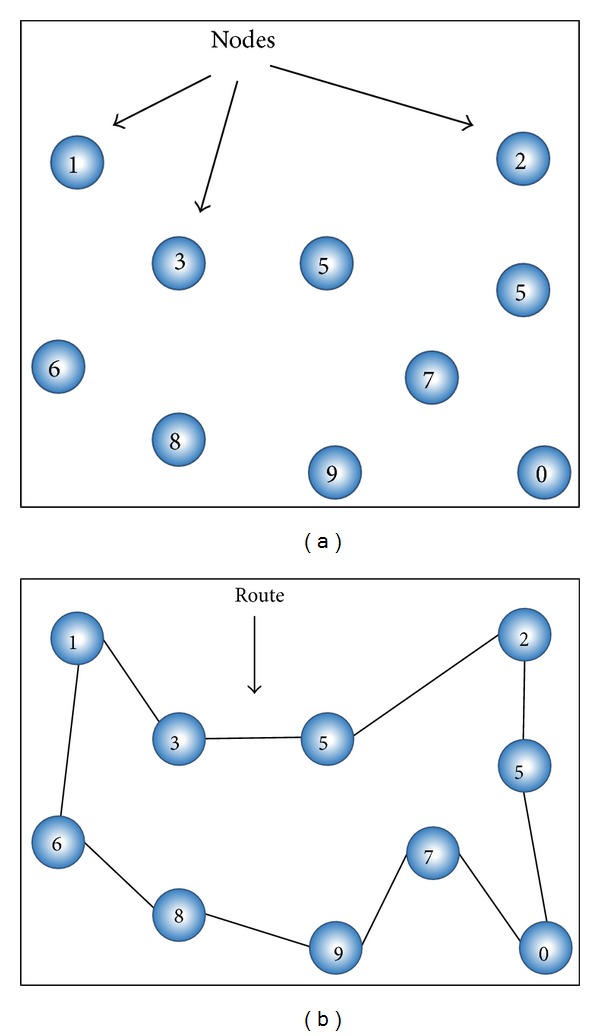
Example of a 10-node ATSP instance and a possible solution.

In this study, the solutions for the ATSP are encoded using the permutation representation [65]. According to this encoding, each solution is represented by a permutation of numbers, which represents the order in which the nodes are visited. For example, for a possible 10-node instance, one feasible solution would be encoded as *X* = (0,5, 2,4, 3,1, 6,8, 9,7), and its fitness would be *f*(*X*) = *d*_05_ + *d*_52_ + *d*_24_ + *d*_43_ + *d*_31_ + *d*_16_ + *d*_68_ + *d*_89_ + *d*_97_ + *d*_70_. This situation is depicted in Figure 4.

### 3.2. Vehicle Routing Problem with Backhauls

The VRPB is a variant of the basic VRP where customers can demand either a delivery or a pick-up of certain goods [57]. In this problem, all deliveries are done before the pick-ups. This is so because, otherwise, it could be a movement of material within the mobile unit that could be counterproductive, for example, putting collected materials on the front of the trunk, whereas at the bottom some goods remain undelivered. The VRPB is widely used in the literature thanks to its applicability to the real world and to its solving complexity [66–68].

The VRPB can be defined as a complete graph *G* = (*V*, *A*), where *V* = {*v*_0_, *v*_1_,…, *v*_*n*_} is the set of vertexes and *A* = {(*v*_*i*_, *v*_*j*_) : *v*_*i*_, *v*_*j*_ ∈ *V*, *i* ≠ *j*} is the set of arcs. The vertex *v*_0_ represents the depot, and the rest are the customers. Besides, in order to facilitate the formulation, the set of customers *V* can be separated into two subsets [69]. The first one, *L*, called* linehaul customers*, contains those users who demand the delivery of goods. On the other hand, the second subset, *B*, called* backhaul customers*, demand the pick-up of a certain amount of material. To express customer demand (*q*_*i*_), positive values are used for linehaul customers and negative values for backhaul ones.

Additionally, a fleet of *K* vehicles is available with a limited capacity *Q*. The objective of the VRPB is to find a number of routes with a minimum cost such that (i) each route starts and ends at the depot, (ii) each client is visited exactly by one route, (iii) all deliveries are made before pick-ups, and (iv) the total demand of the customers visited by one route does not exceed the total capacity of the vehicle that performs it.

**Figure 5:**
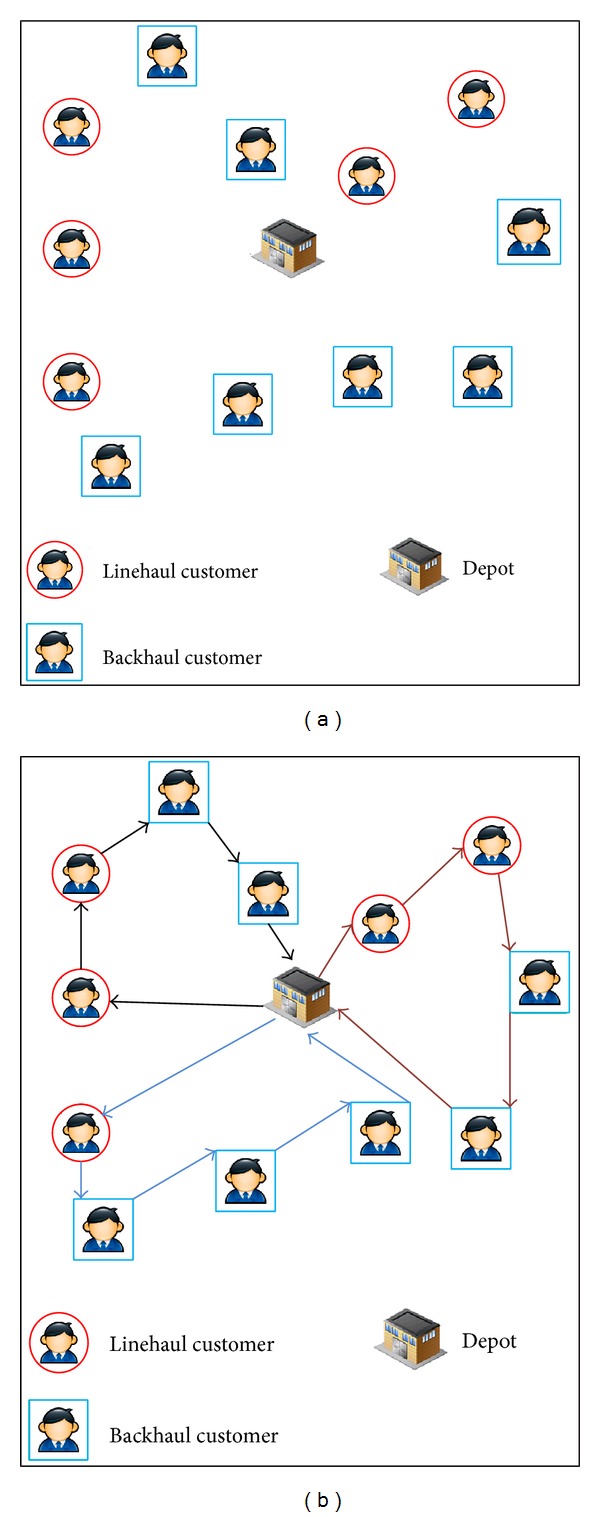
Example of a VRPB instance and a possible solution.

Finally, the permutation representation is also used for this problem [70], and the routes are also encoded as nodes permutation. In addition, to distinguish the different routes in a solution, they are separated by zeros. As an example, suppose a set of five linehaul customers *L* = {*L*1, *L*2, *L*3, *L*4, *L*5} and seven backhaul customers *B* = {*B*1, *B*2, *B*3, *B*4, *B*5, *B*6, *B*7}. One possible solution with three vehicles would be *X* = (*L*5, *L*3, *B*1, *B*6, 0, *L*4, *L*1, *B*3, *B*7, 0, *L*2, *B*4, *B*5, *B*2) and its fitness would be *f*(*X*) = *d*_0*L*5_ + *d*_*L*5*L*3_ + *d*_*L*3*B*1_ + *d*_*B*1*B*6_ + *d*_*B*60_ + *d*_0*L*4_ + *d*_*L*4*L*1_ + *d*_*L*1*B*3_ + *d*_*B*3*B*7_ + *d*_*B*70_ + *d*_0*L*2_ + *d*_*L*2*B*4_ + *d*_*B*4*B*5_ + *d*_*B*5*B*2_ + *d*_*B*20_. In Figure 5(a), an example of a VRPB instance is depicted. Furthermore, in Figure 5(b), a possible solution for this instance is shown.

### 3.3. n-Queen Problem

The NQP is a generalization of the problem of putting eight nonattacking queens on a chessboard [71], which was introduced by Bezzel in 1848 [72]. The NQP consists in placing *N* queens on a *N* × *N* chessboard, in order that they cannot attack each other. This problem is a classical combinatorial design problem (constraint satisfaction problem), which can also be formulated as a combinatorial optimization problem [73]. In this paper, the NQP has been formulated as a combinatorial optimization problem, where a solution *X* is coded as an *N*-tuple (*q*_1_, *q*_2_,…, *q*_*n*_), which is a permutation of the set (1,2,…, *N*). Each *q*_*i*_ represents the row occupied by the queen positioned in the *i*th column. Using this representation, vertical and horizontal collisions are avoided, and the complexity of the problem becomes *O*(*N*!). Thereby, the fitness function is defined as the number of diagonal collisions along the board. Notice that *i*th and *j*th queens collide diagonally if

(5) $$\begin{array}{c} \left|i-q_{i}\right|=\left|j-q_{j}\right|\;\forall i,j:\left\{1,2,\ldots ,N\right\};i\neq j. \end{array}$$

**Figure 6:**
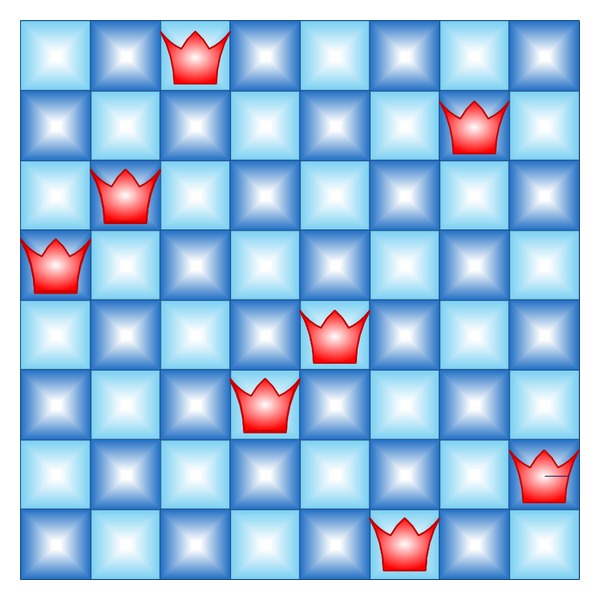
Example of an 8 × 8 instance for the NQP.

In this way, the objective of NQP is to minimize the number of conflicts, the ideal fitness being zero. A possible solution for an 8-queen chessboard is depicted in Figure 6. According to the encoding explained, the solution represented in this figure would be encoded as *f*(*X*) = (4,3, 1,6, 5,8, 2,7). Additionally, its fitness would be 3, since there are three diagonal collisions (4-3, 6-5, and 6–8). This same formulation has been used before in the literature [74, 75].

### 3.4. One-Dimensional Bin Packing Problem

The packing of items into boxes or bins is a daily task in distribution and production. Depending on the item characteristics, as well as the form and capacity of the bins, a wide amount of different packing problems can be formulated. In [59], an introduction to bin-packing problems can be found. The BPP is the simplest one, and it has been frequently used as a benchmarking problem [76–78]. The BPP consists of a set of items *I* = (*i*_1_, *i*_2_,…, *i*_*n*_), each with an associated size *s*_*i*_ and an unlimited supply of bins with the same capacity *q*. The objective of the BPP is to pack all the items into a minimum number of bins. In this way, the objective function is the number of bins, which has to be minimized.

**Figure 7:**
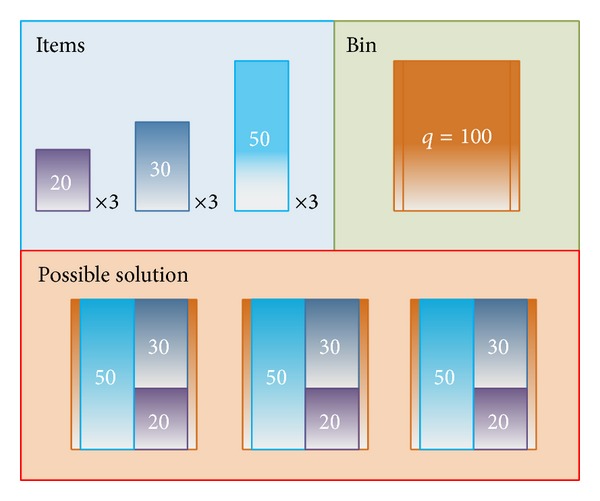
Example of a BPP instance and a possible solution.

In this study, the solutions are encoded as a permutation of items. To count the number of bins needed in one solution, the item sizes are accumulated in a variable (*sumSize*). When *sumSize* exceeds *q*, the number of bins is incremented in 1, and *sumSize* is reset to 0. Thereby, we suppose a simple instance of 9 items *I* = {*i*_1_, *i*_2_,…, *i*_9_}, three different sizes *s*_1−3_ = 20, *s*_4−6_ = 30, and *s*_7−9_ = 50, and *q* = 100. One possible solution could be *X* = (*i*_1_, *i*_4_, *i*_7_, *i*_2_, *i*_5_, *i*_8_, *i*_3_, *i*_6_, *i*_9_), and its fitness would be 3 (the number of bins needed to hold all the items). This example is represented in Figure 7.

## 4. Experimentation Setup

In this section, the experimentation performed is described. According to the study carried out in [51], the GB metaheuristic provides some originality regarding the well-known techniques that can be found today in the literature. In line with this, analyzing the philosophy and the working way of GB, it can be concluded that the DGA is the technique which shares the most similarities with it. Among other similarities, in the evolution of their individuals, these metaheuristics rely on two operators, a local and a cooperative one, which are used for the exploitation and exploration. In addition, these techniques are easy to apply to combinatorial optimization problems.

For these reasons, to prove the quality of the GB, two single-population GAs and two DGAs are used for the experimentation. The general characteristics of these four techniques are explained in Section 4.1. In addition, the parametrization of all the algorithms is described in the same section. The details of the experimentation are introduced in Section 4.2.

### 4.1. General Description of Developed Techniques

As has been mentioned, the outcomes obtained by the GB are compared with the ones obtained by two basic single-population GAs (GA_1_ and GA_2_) and two different DGAs (DGA_1_ and DGA_2_). The structure used for both GAs is the one represented in Algorithm 1, and it is considered the conventional one. Furthermore, in Algorithm 2, the structure of both DGAs is depicted, which is also the conventional one.

On one hand, for GA_1_ and DGA_1_ conventional operators and parameters have been used, that is, a high crossover probability and a low mutation probability. These concepts are based on the concepts outlined in many previous studies [54, 79, 80]. On the other hand, for GA_2_ and DGA_2_, parameters have been adjusted to be similar to those used for the GB. Thereby, the numbers of cooperative movements (crossovers and custom trainings) and individual movements (mutations and conventional trainings) performed are the same. In addition, the same functions have been used for GA_2_, DGA_2_, and GB. In this way, the only difference between them is the structure. Thereby, it can be deduced which algorithm is the one that obtains better results, using the same operators for the same number of times.

The population size used for each metaheuristic is 48. All the initial solutions have been generated randomly. For DGA_1_ and DGA_2_, this population has been divided into 4 subpopulations of 12 individuals. For the GB, the whole population is also divided into 4 teams of 12 players each. The crossover probability (*p*_*c*_) and mutation probability (*p*_*m*_) of the GA_1_ are 95% and 5%, respectively. On the other hand, different *p*_*c*_ and *p*_*m*_ have been used for every subpopulation of DGA_1_. For *p*_*c*_, 95%, 90%, 80%, and 75% have been utilized, and for *p*_*m*_, 5%, 10%, 20%, and 25% have been utilized. At last, for GA_2_ and DGA_2_, *p*_*c*_ = 0.003% and *p*_*m*_ = 100% have been used, in order to fit with the GB parameters.

In relation to the parents selection criteria for the GAs and DGAs, first, each individual of the population is selected as parent with a probability equal to *p*_*c*_. If one individual is selected for the crossover, the other parent is selected randomly. Regarding the survivor function, a 100% elitist function has been developed for GA_2_ and DGA_2_, and a 50% elitist random (which means that the half of the survivor population is composed of the best individuals, and the remaining ones are selected randomly) has been developed for GA_1_ and DGA_1_. In DGA_1_ and DGA_2_, the classic best-replace-worst migration strategy has been used. In this strategy, every subpopulation *i* shares its best individual with the following *i* + 1 deme, in a ring topology. This communication happens every generation and the immigrant replaces the worst individual of deme *i* + 1. Ultimately, the execution of both GAs and DGAs finishes when there are *n* + ∑_*k*=1_^*n*^ *k* generations without improvements in the best solution, where *n* is the size of the problem. The problem size is the number of customers in the two routing problems, the number of queens in the NQP and the number of items in the BPP.

The successor functions employed by GB as conventional training functions for the ATSP, NQP, and BPP are the following.

- 2-opt and 3-opt: these functions, proposed by Lin for the TSP [60], have been used widely in the literature [81–83]. These operators eliminate at random 2 (for the 2-opt) and 3 (for the 3-opt) arcs of the solution, and they create two or three new arcs, avoiding the generation of cycles.
- Insertion function: this operator selects and extracts one random node of a solution, and it inserts it in another random position. Because of its easy implementation and its good performance, this function is often used in the literature for any kind of permutation encoded problem [84, 85].
- Swapping function: this well-known function is also widely employed in lots of research studies [86]. In this case, two nodes of a solution are selected randomly, and they swap their position.

These successor functions have also been used as mutation functions for the DGA_2_ (a different function for each subpopulation). On the other hand, for the GA_1_, GA_2_, and DGA_1_, the* 2-opt* has been utilized for this purpose, since it is the one that gets the best results.

**Figure 8:**
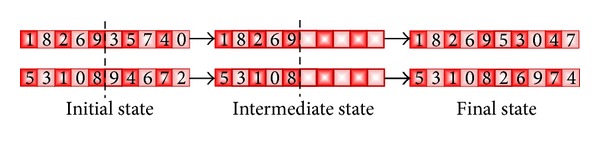
Example of HX with a 10-node instance.

**Figure 9:**
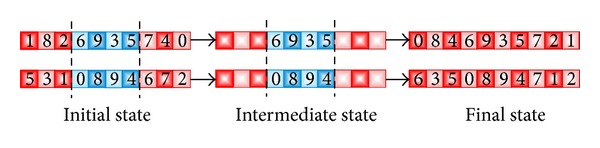
Example of OX with a 10-node instance.

For the same problems (ATSP, NQP, and BPP), GB uses the half crossover (HX) operator [81] as a custom training function. This operator is a particular case of the traditional one-point crossover, in which the cut-point is made always in the middle of the solution. Assuming a 10-node instance, in Figure 8, an example of this function can be seen. This function has been used as a crossover operator for the GA_2_ and DGA_2_. On the other hand, for the GA_1_ and DGA_1_, the well-known order crossover (OX) [61] has been implemented as a crossover function. In Figure 9, an example of the OX is shown. Finally, in Table 1, a summary of the characteristics of both GAs and DGAs is depicted.

Regarding the VRPB,* 2-opt* and* Insertion* functions are also used as conventional training functions. These operators, as Savelsbergh called them [87], are intraroute functions, which means that they work within a specific route. Additionally, two interroute functions have been developed.

- Insertion Routes: this function selects and extracts one random node from a random route. After that, this node is reinserted in a random position in another randomly selected route. This function could create new routes.
- Swapping Routes: this operator selects randomly two nodes of two randomly selected routes. These nodes are swapped.

It is noteworthy that all these functions take into account both the vehicles capacity and the class of the nodes' demands, never making infeasible solutions. As in the previous problems, these same operators have been also developed as mutation functions for the DGA_2_ (a different operator for each subpopulation). Moreover,* Insertion Routes* operator has been used for the same purpose in GA_1_, GA_2_, and DGA_1_.

**Figure 10:**
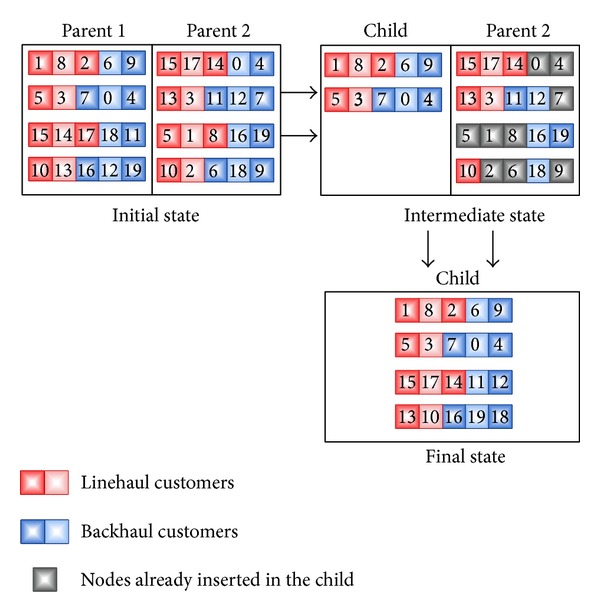
A possible example of HRX with a 20-node instance.

For the VRPB, the half route crossover (HRX) has been used as custom training. This function has been used recently in several studies [49, 85], and it operates as follows: first, half of the routes of one parent are directly inserted into the child. Then, the nodes that remain to be inserted are added in the same order in which they appear in the other parent. As the above functions, the HRX takes into account the VRPB constraints, and it does not generate infeasible solutions. In Figure 10, an example of the HRX procedure in a 20-node instance is shown. Additionally, in Table 2, a summary of the characteristics of both GAs and DGAs for VRPB is shown. Finally, the features of the GB for the four problems are depicted in Table 3.

### 4.2. Description of the Experimentation

In this section, the basic aspects of the experimentation are detailed. First, all the tests have been run on an Intel Core i7 3930 computer, with 3.20 GHz and a RAM of 16 GB. Microsoft Windows 7 has been used as OS. All the techniques were coded in Java. For the ATSP, 19 instances have been employed, which have been obtained from the TSPLib Benchmark [88]. These 19 instances are all the available ones that can be found in the TSPLib webpage (https://www.iwr.uni-heidelberg.de/groups/comopt/software/TSPLIB95/). Additionally, 12 instances have been utilized for the VRPB. These instances have been created by the authors of this study. With the aim of allowing the replication of this experimentation, the benchmark used is available under request, and it can be obtained from the personal site of the corresponding author of this paper (http://paginaspersonales.deusto.es/e.osaba). The first 6 instances of the benchmark have been picked from the VRPTW Solomon Benchmark (http://w.cba.neu.edu/~msolomon/problems.htm). For these instances, the time constraints have been removed. Furthermore, the demands type has been modified in order to create backhaul and linehaul customers. The vehicles capacity and the amount of customer demands have been retained. On the other hand, the remaining instances have been taken from the VRPWeb, and they belong to the Christofides/Eilon (http://neo.lcc.uma.es/vrp) CVRP set. In this case, only the demand nature has been modified. These problem instances have been adapted for experimentation purpose and so, their optimum solutions are unknown.

In regard to the NQP, 15 different instances have been developed. The name of each of them describes the number of queens and the dimension of the chessboard. For example, the 20-queen instance consists in placing 20 queens on a 20x20 board. For this problem, the optimum is also not shown, since it is 0 in every case. At last, regarding the BPP, 16 instances have been chosen from the well-known Scholl/Klein benchmark (http://www.wiwi.uni-jena.de/entscheidung/binpp/index.htm). These cases are named *NxCyWz*_*a*, where *x* is 1 (50 items), 2 (100 items), 3 (200 items), or 4 (500 items); *y* is 1 (capacity of 100), 2 (capacity of 120), and 3 (capacity of 150); *z* is 1 (items size between 1 and 100) and 2 (items size between 20 and 100); and *a* is A, B, or C as benchmark indexing parameter.

Each instance has been run 40 times. Besides, with the intention of conducting a fair and rigorous outcomes' comparison, two different statistical tests have been performed: the normal distribution *z*-test and the Friedman test. Thanks to these tests, it can be concluded whether the differences in the results are statistically significant or not. The details of these statistical tests are explained in the next section.

## 5. Experimentation Results

In this section, the results obtained by each technique for the chosen problems are shown and analysed. In addition, the statistical tests are also depicted in this section. First, the results and statistical tests are displayed (Section 5.1). Then, the analysis of the outcomes obtained is conducted (Section 5.2).

### 5.1. Results and Statistical Tests

In this section, the outcomes and statistical tests are shown. In Table 4, the results obtained for the ATSP are introduced. Furthermore, in Table 5, the outcomes got for the VRPB are presented. Besides, results obtained for the NQP and BPP are detailed in Tables 6 and 7, respectively. For each instance, the average result and standard deviation are shown. Additionally, average runtimes are also displayed (in seconds).

As mentioned, two statistical tests have been performed according to these outcomes. The first one is the normal distribution *z*-test. By this test, the results obtained by the GB are compared with those obtained by the other techniques. Thanks to the normal distribution *z*-test, it can be concluded whether the differences between GB and the other techniques are statistically significant or not. The *z* statistic has the following form:

(6) $$\begin{array}{c} z=\frac{\bar{X_{\text{GB}}}-\bar{X_{i}}}{\sqrt{\left(\sigma_{\text{GB}}^{2}/n_{\text{GB}}\right)+\left(\sigma_{i}^{2}/n_{i}\right)}}, \end{array}$$

where $\bar{X_{\text{GB}}}$: average of the GB, *σ*_GB_: standard deviation of the GB, *n*_GB_: sample size for GB, $\bar{X_{i}}$: average of the technique *i*, *σ*_*i*_: standard deviation of the technique *i*, and *n*_*i*_: sample size for technique *i*.

It is noteworthy that the GB has been faced with the other four implemented metaheuristics. Thereby, the parameter *i* can be GA_1_, GA_2_, DGA_1_, and DGA_2_. The confidence interval has been stated at 95% (*z*_0.05_ = 1.96). In this way, the result of the test can be positive (+), if *z* ≥ 1.96; negative (−), if *z* ≤ −1.96; or neutral (∗), if −1.96 < *z* < 1.96. A + indicates that GB is significantly better. In the opposite case, it obtains substantially worse solutions. If the result is (∗), the difference is not significant. In this study, the numerical value of *z* is also displayed. Thereby, the difference in results may be seen more easily. In Table 8, the tests performed for the chosen problems are shown.

The second statistical test conducted is the Friedman test. In Table 9, the results of overall ranking calculated using this test are summarized, where the smaller the score is, the better the ranking is. This ranking is conducted considering the average results of each technique and comparing them instance by instance. Furthermore, in order to check if there are statistical differences between the developed techniques, the value of *X*_*r*_^2^ is also depicted in Table 9. This value has been obtained using the following formula:

(7) $$\begin{array}{c} X_{r}^{2}=\frac{12}{HK\left(K+1\right)}\sum {\left(HRc\right)}^{2}-3H\left(K+1\right). \end{array}$$

*H* is the number of problem instances (e.g., for ATSP, *H* = 19), *K* is the number of techniques (*K* = 5), and *Rc* is the value of the Friendman test ranking score. The confidence interval has been stated at the 99% confidence level. The critical point in a *χ* ^2^ distribution with 4 degrees of freedom is 13.277. Thereby, if *X*_*r*_^2^ > 13.277, it can be concluded that there are significant differences between the five techniques. Otherwise, the differences are not substantial.

### 5.2. Analysis of the Results

Looking at the results presented in the previous section, one clear conclusion can be drawn: the GB outperforms the other techniques in terms of quality. Analyzing Tables 4–7, it can be seen how GB obtains better results than the other metaheuristics in 95.16% of the instances (59 out of 62). In the remaining 3 instances, GB obtains the same outcomes as one or more of the other techniques. Besides, as can be proved, GB has never obtained worse results. In addition, as Table 8 demonstrates, GB obtains significantly better results in 95.96% of the cases (238 out of 248), the differences being insignificant in the remaining 4.04%. The conclusions that can be extracted by performing a problem-by-problem analysis are the same. Regarding the ATSP, the GB gets better results in 94.73% of the cases (18 out of 19). In the remaining instance, GB obtains the same results as DGA_1_ and DGA_2_. Furthermore, according to Table 8, GB is substantially better in 97.36% of the confrontations (74 out of 76). In regard to VRPB and BPP, GB outperforms the other alternatives in 100% of the cases, and the differences are significant in 93.75% (45 out of 48) of the confrontations for the VRPB and in 100% (64 out of 64) for the BPP. Finally, in relation to NQP, GB proves to be better in 86.66% of the instances. In the remaining 2 cases, it obtains the same results as one or more of the other techniques. Besides, these differences are significantly better for the GB in 91% of the confrontations (55 out of 60).

At last, observing the results offered by the Friedman test (Table 9), it can be seen how GB is arguably the best technique for all the problems. In addition, all the values of *X*_*r*_^2^ are higher than the critical point, 13.277. For this reason, it can be concluded again that there are significant differences among the results obtained by the four techniques for all the problems.

The reasons why GB performs better than the other algorithms are the same that were presented in [51]. On the one hand, the GB combines local improvement phases (conventional trainings) with cooperative (custom trainings and player transfers) and competitive phases (matches). This technique gives greater importance to the autonomous improvement of the players, while the other four algorithms are more focused on the cooperation and competition of individuals. Furthermore, GB uses cooperation between players through custom trainings. Anyway, this resource is used to avoid local optima and to increase the exploration capacity of the technique. For this reason, this kind of trainings is used sporadically and only when it is beneficial for the search process. Besides, in the GB metaheuristic, players can explore different neighborhood structures. This feature is another alternative to avoid local optima, and it helps players to explore in different ways the solution space. On the other hand, GA_1_, GA_2_, DGA_1_, and DGA_2_ have also some mechanisms to avoid local optima, but optimization mechanisms are not as powerful as the GB.

Regarding runtimes, GB is faster than GA_1_ and DGA_1_, while GA_2_ and DGA_2_ need similar times to GB. This fact gives an advantage to GB, since it can obtain better results than the rest of techniques needing similar runtimes as GA_2_ and DGA_2_.

The reason why GB is faster than GA_1_ and DGA_1_ can be explained following the same concepts introduced in several recently published works [49, 81]. Comparing individual improvement operators (mutation and custom training) and cooperative operators (crossover and custom training), the first ones need less time. They operate with one solution, and they perform a simple modification which can be made in a minimum time. On the other hand, cooperative operators work with two different solutions, and their working ways are more complex, needing more runtime. GB makes less cooperative movements than GA_1_ and DGA_1_, and this fact is perfectly reflected in runtimes. Additionally, GB, GA_2_, and DGA_2_ obtain similar runtimes because they use their operators similarly.

Another noteworthy fact is the robustness of the GB. The standard deviation of the GB is lower than the one of the other techniques in 93.54% of the instances (58 out of 62). This means that the differences between the worst and the best result found for an instance are lower for the GB, in comparison with the four other algorithms. This fact provides robustness and reliability to the metaheuristic, something very important for a real-world problem.

As a final conclusion, it can be drawn that the GB has proved to be better than the other four metaheuristics for all the used problems. In this way, adding these outcomes to those presented in [51] for the TSP and CVRP, it can be confirmed that the GB is a promising technique for solving combinatorial optimization problems.

## 6. Conclusions and Further Work

The Golden Ball is a recently published multiple-population metaheuristic, which is based on soccer concepts. Until now, its performance has been tested with two simple routing problems, the TSP and the CVRP. In this paper, the quality of this technique is demonstrated applying it to four additional combinatorial problems. Two of them are routing problems, which are more complex than the previously used ones: the ATSP and the VRPB. Furthermore, one constraint satisfaction problem (NQP) and one combinatorial design problem (BPP) have also been used. In the paper presented, GB has been tested with 62 new problem instances. The outcomes obtained by the GB have been compared with the ones got by two different GAs and two DGAs. Additionally, two statistical tests have been conducted, in order to perform a rigorous and fair comparison. As a conclusion, adding the results obtained in this study with those obtained in [51], it can be concluded that the GB is a promising metaheuristic to solve combinatorial optimization problems.

As future work, it has been planned to apply the GB to other types of optimization problems. In addition, it is intended to compare the technique with other population metaheuristics, in terms of concepts and results.

## Figures and Tables

**Algorithm 1:**
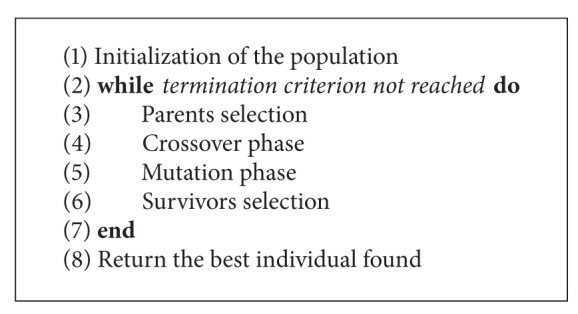
Pseudocode of GA_1 _and GA_2_.

**Algorithm 2:**
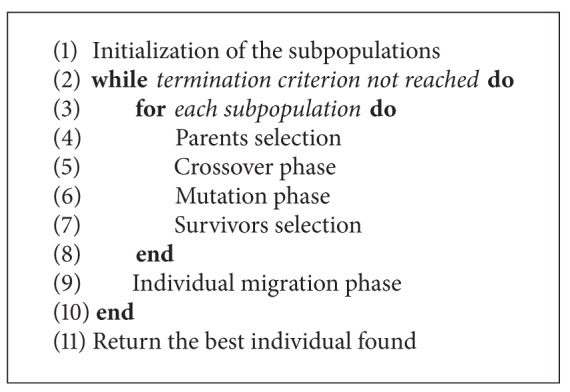
Pseudocode of DGA_1 _and DGA_2_.

**Table 1:** Summary of the characteristics of GA_1_, GA_2_, DGA_1_, and DGA_2_ for the ATSP, NQP, and BPP.

Alg.	Population	*p*_*c*_ and *p*_*m*_	Surviv. funct.	Cross. oper.	Mut. operators
GA_1_	1 population, 48 individuals	80% & 20%	50% elitist—50% random	OX	2-opt
GA_2_	1 population, 48 individuals	0.003% & 100%	100% elitist	HX	2-opt
DGA_1_	4 subpopulations, each with 12 individuals	95% & 5%, 90% & 10%, 75% & 25%, 80% & 20%, respectively	50% elitist—50% random	OX	2-opt (the same for all subpopulations)
DGA_2_	4 subpopulations, each with 12 individuals	0.003% & 100%	100% elitist	HX	2-opt, 3-opt, Swapping, and Insertion (a different function for each population)

**Table 2:** Summary of the characteristics of GA_1_, GA_2_, DGA_1_, and DGA_2_ for the VRPB.

Alg.	Population	*p*_*c*_ and *p*_*m*_	Surviv. funct.	Cross. oper.	Mut. operators
GA_1_	1 population, 48 individuals	80% & 20%	50% elitist—50% random	HRX	Insertion Routes
GA_2_	1 population, 48 individuals	0.003% & 100%	100% elitist	HRX	Insertion Routes
DGA_1_	4 subpopulations, each with 12 individuals	95% & 5%, 90% & 10%, 75% & 25%, 80% & 20%, respectively	50% elitist—50% random	HRX	Insertion Routes (the same for all subpopulations)
DGA_2_	4 subpopulations, each with 12 individuals	0.003% & 100%	100% elitist	HRX	2-opt, Insertion, Insertion Routes, and Swapping Routes (a different function for each population)

**Table 3:** Summary of the characteristics of GB.

Number of teams (TN)	4
Number of players per team (PT)	12
Number of trainings without improvement for a *custom training *	6
Number of trainings without improvement for a *special transfer *	12
Custom training function for the ATSP, NQP, and BPP	HX
Custom training function for the VRPB	HRX
Conventional training functions for the ATSP, NQP, and BPP	2-opt, 3-opt, Swapping, and Insertion
Conventional training functions for the VRPB	2-opt, Insertion, Swapping Routes, and Insertion Routes

**Table 4:** Results of GB, GA_1_, GA_2_, DGA_1_, and DGA_2_ for the ATSP. For each instance results average, standard deviation, and time average are shown.

Instance		GB			GA_1_			GA_2_			DGA_1_			DGA_2_		
Name	Optimum	Avg.	S. dev.	Time	Avg.	S. dev.	Time	Avg.	S. dev.	Time	Avg.	S. dev.	Time	Avg.	S. dev.	Time
br17	39	**39.0**	0.0	0.1	39.2	0.4	0.1	39.1	0.2	0.1	**39.0**	0.0	0.1	**39.0**	0.0	0.2
ftv33	1286	**1329.2**	33.7	0.2	1412.5	81.5	0.4	1540.3	83.1	0.2	1403.7	60.9	0.4	1416.8	90.4	0.4
ftv35	1473	**1509.5**	28.8	0.2	1609.1	76.9	0.4	1678.3	165.3	0.2	1606.8	74.7	0.4	1598.3	57.0	0.4
ftv38	1530	**1580.4**	37.3	0.3	1676.1	71.7	0.5	1709.1	145.8	0.3	1703.6	91.8	0.5	1699.4	74.5	0.4
p43	5620	**5620.6**	0.8	0.3	5627.7	5.5	0.9	5626.9	3.8	0.4	5625.9	3.7	0.8	5624.8	3.4	0.4
ftv44	1613	**1695.1**	42.7	0.4	1787.1	93.2	1.0	2071.5	147.7	0.4	1832.6	131.9	0.9	1835.0	108.0	0.6
ftv47	1776	**1862.2**	55.2	0.5	1961.4	86.7	1.4	2526.2	705.5	0.6	2020.2	139.1	1.0	2038.2	130.7	0.8
ry48p	14422	**14614.2**	164.5	0.6	15008.2	348.6	1.6	14976.5	259.7	0.8	15038.8	381.9	1.8	14945.2	178.8	0.7
ft53	6905	**7335.0**	204.7	0.8	8077.2	344.9	1.8	9401.1	632.6	0.9	8331.5	462.9	1.7	7997.4	232.2	0.9
ftv55	1608	**1737.1**	73.2	0.8	1879.3	110.7	1.4	2152.4	312.5	1.4	2021.2	153.4	1.7	1990.9	109.4	1.4
ftv64	1839	**2023.5**	93.4	1.6	2203.5	129.5	2.1	3032.9	226.8	1.8	2284.3	163.2	3.2	2321.8	141.3	1.7
ftv70	1950	**2151.9**	83.9	1.8	2313.7	145.2	2.7	3335.5	330.2	2.1	2390.0	127.0	2.5	2509.6	140.4	2.1
ft70	38673	**40135.9**	461.4	2.1	40416.0	623.4	3.2	47067.0	1647.2	2.1	40813.1	746.0	2.6	41129.9	823.5	2.3
kro124p	36230	**38924.6**	1157.4	7.4	42259.0	1813.8	9.4	44084.0	1932.5	8.8	43408.1	2020.3	11.4	41116.5	1044.9	7.8
ftv170	2755	**3873.4**	468.7	41.2	4214.8	361.8	49.8	4210.1	481.3	43.5	4367.0	470.7	51.7	4252.4	174.2	39.8
rbg323	1326	**1494.2**	35.7	120.3	1601.0	76.8	130.7	1596.1	77.3	124.9	1584.7	73.7	130.7	1614.7	194.4	124.9
rbg358	1163	**1364.8**	40.1	147.7	1781.9	62.5	158.1	1799.8	66.2	150.4	1720.8	175.0	164.8	1724.7	189.7	159.4
rbg403	2465	**2510.4**	29.6	222.0	3088.4	199.6	227.4	3298.8	378.1	224.2	2870.2	194.5	235.1	2766.2	138.4	220.4
rbg443	2720	**2767.9**	17.5	324.5	3142.5	219.3	335.9	3154.4	242.5	321.0	2992.2	125.6	335.9	2989.6	128.1	329.0

**Table 5:** Results of GB, GA_1_, GA_2_, DGA_1_, and DGA_2_ for the VRPB. For each instance results average, standard deviation, and time average are shown.

Instance	GB			GA_1_			GA_2_			DGA_1_			DGA_2_		
Name	Avg.	S. dev.	Time	Avg.	S. dev.	Time	Avg.	S. dev.	Time	Avg.	S. dev.	Time	Avg.	S. dev.	Time
C101	**675.3**	39.1	3.3	722.3	67.7	10.4	706.0	40.2	2.9	739.1	47.7	12.6	707.0	65.6	3.4
C201	**648.6**	44.1	1.1	852.3	124.5	1.2	834.8	75.3	1.4	795.8	50.2	2.4	717.2	133.7	1.1
R101	**895.8**	25.1	3.1	995.8	80.9	7.8	946.1	48.8	2.5	959.5	43.9	9.1	903.9	51.5	3.1
R201	**1047.6**	22.6	7.0	1270.0	62.5	13.0	1137.2	65.4	6.5	1188.9	75.1	12.4	1085.7	39.5	6.8
RC101	**583.3**	15.1	0.5	778.9	118.9	0.9	660.2	59.2	0.9	645.1	66.3	1.1	626.3	44.1	1.1
RC201	**1164.6**	41.6	6.2	1304.5	76.5	13.2	1261.0	87.9	5.9	1337.2	60.1	12.4	1182.1	63.8	6.1
En23k3	**696.8**	13.5	0.5	797.0	67.8	0.9	748.4	33.9	0.8	771.0	49.3	0.8	702.6	24.1	0.8
En30k4	**509.6**	16.3	0.5	672.2	51.7	1.5	630.7	39.7	1.3	600.6	56.6	1.4	593.3	69.2	0.8
En33k4	**777.9**	30.7	0.6	851.7	41.9	1.7	835.7	47.3	1.1	833.6	35.3	1.2	819.7	28.7	0.9
En51k5	**630.5**	20.7	2.0	716.8	52.3	2.6	715.0	46.5	2.3	721.5	33.5	2.5	646.0	35.6	1.9
En76k8	**830.7**	26.4	6.3	915.2	43.1	10.7	916.3	54.3	6.1	918.5	74.0	9.7	871.4	39.2	5.8
En101k14	**1088.0**	24.2	22.0	1183.8	38.8	26.3	1164.8	56.2	20.8	1231.9	42.9	24.8	1191.4	59.1	19.8

**Table 6:** Results of GB, GA_1_, GA_2_, DGA_1_, and DGA_2_ for the NQP. For each instance, results average, standard deviation, and time average are shown. The optimum of each instance is 0.

Instance	GB			GA_1_			GA_2_			DGA_1_			DGA_2_		
Name	Avg.	S. dev.	Time	Avg.	S. dev.	Time	Avg.	S. dev.	Time	Avg.	S. dev.	Time	Avg.	S. dev.	Time
8-queen	**0.0**	0.0	0.1	**0.0**	0.0	0.1	**0.0**	0.0	0.1	**0.0**	0.0	0.1	**0.0**	0.0	0.1
20-queen	**0.1**	0.2	0.1	1.4	0.6	0.1	**0.1**	0.3	0.1	1.5	1.1	0.2	0.8	0.7	0.1
50-queen	**0.0**	0.0	0.7	5.3	1.7	0.8	1.9	0.7	0.8	5.0	1.1	1.1	4.3	1.6	0.8
75-queen	**0.1**	0.2	4.1	8.1	1.6	4.1	4.6	1.8	4.6	9.1	1.7	5.4	6.1	1.7	4.8
100-queen	**0.5**	0.7	5.8	13.6	2.1	6.8	7.2	1.7	7.2	12.0	2.0	10.1	11.4	3.0	11.0
125-queen	**0.3**	0.4	13.4	16.4	3.2	15.8	12.6	2.4	14.8	16.2	2.5	18.4	14.3	2.4	14.8
150-queen	**1.7**	1.4	16.7	18.1	3.2	18.4	17.0	2.9	16.5	20.0	3.2	20.6	19.0	1.9	16.5
200-queen	**3.3**	1.9	23.1	26.0	3.9	26.1	24.5	3.5	26.1	32.8	4.8	31.1	23.4	3.1	26.1
225-queen	**4.3**	1.7	35.4	31.9	5.0	41.5	37.9	3.2	31.2	38.4	3.5	31.2	29.2	4.3	35.8
250-queen	**3.5**	1.6	72.4	44.3	3.9	83.1	32.7	6.7	78.1	41.2	5.3	78.1	32.0	3.1	78.1
275-queen	**5.6**	3.0	101.6	50.0	11.2	104.2	39.5	4.9	102.5	44.1	7.5	107.6	39.9	4.9	104.7
300-queen	**6.4**	2.6	131.0	61.9	5.2	132.9	44.4	5.3	130.9	52.8	5.9	134.5	44.4	5.9	128.4
325-queen	**4.8**	2.4	215.6	63.5	5.6	225.3	47.4	6.4	220.7	54.4	3.6	228.7	49.1	4.1	218.1
350-queen	**5.1**	3.0	275.3	71.4	5.6	286.7	51.0	4.7	281.2	65.5	5.7	289.6	49.9	5.8	278.5
400-queen	**4.3**	2.2	359.7	59.9	10.1	371.8	54.0	9.7	365.7	59.4	8.1	379.5	56.1	7.6	357.8

**Table 7:** Results of GB, GA_1_, GA_2_, DGA_1_, and DGA_2_ for the BPP. For each instance results average, standard deviation, and time average are shown.

Instance		GB			GA_1_			GA_2_			DGA_1_			DGA_2_		
Name	Optimum	Avg.	S. dev.	Time	Avg.	S. dev.	Time	Avg.	S. dev.	Time	Avg.	S. dev.	Time	Avg.	S. dev.	Time
N1C1W1_A	25	**26.0**	0.0	0.2	26.5	0.5	0.2	26.7	0.4	0.1	26.8	0.5	0.3	26.7	0.5	0.3
N1C1W1_B	31	**31.0**	0.0	0.2	31.9	0.4	0.2	31.5	0.5	0.2	31.5	0.5	0.4	31.6	0.6	0.3
N1C2W1_A	21	**21.1**	0.2	0.2	21.9	0.5	0.3	21.9	0.5	0.2	21.8	0.4	0.4	22.0	0.4	0.3
N1C2W1_B	26	**26.1**	0.2	0.3	27.6	0.5	0.3	27.1	0.4	0.3	26.8	0.4	0.3	26.8	0.5	0.3
N2C1W1_A	48	**51.0**	0.3	1.8	53.1	0.6	1.7	52.4	0.6	1.4	52.9	0.6	1.8	52.2	0.7	1.4
N2C1W1_B	49	**51.4**	0.5	1.8	52.6	0.6	1.9	53.0	0.8	1.5	53.3	0.8	1.8	52.8	0.6	1.4
N2C2W1_A	42	**43.9**	0.2	1.8	44.6	0.6	1.8	45.4	0.5	1.7	45.7	0.6	1.9	45.3	0.6	1.7
N2C2W1_B	50	**51.4**	0.5	2.1	52.4	0.6	1.9	53.1	0.7	1.8	53.4	0.6	1.9	53.2	0.6	1.5
N3C2W2_A	107	**114.1**	1.1	15.0	121.8	1.3	14.8	118.7	1.5	13.5	120.0	1.4	15.2	118.0	1.3	14.1
N3C2W2_B	105	**109.6**	0.5	17.1	119.8	1.5	16.5	113.4	1.1	16.1	115.3	1.8	15.4	111.9	0.7	14.9
N3C3W1_A	66	**70.2**	0.5	12.2	74.6	0.7	12.9	71.5	0.7	12.1	72.6	0.9	14.8	71.4	0.8	13.8
N3C3W1_B	71	**76.1**	0.5	12.1	78.4	0.6	13.1	77.4	0.9	12.7	78.6	1.0	15.7	77.6	1.0	14.5
N4C1W1_A	240	**260.5**	1.5	194.7	271.6	2.5	187.4	268.4	3.8	181.0	270.1	2.4	200.7	267.7	2.1	199.9
N4C2W1_A	210	**231.2**	1.2	195.8	239.1	1.6	188.5	233.3	5.2	186.4	241.0	1.9	203.2	235.4	1.3	200.1
N4C2W1_B	213	**233.3**	1.6	190.5	241.5	2.4	186.2	234.3	4.7	184.2	243.6	1.9	198.6	239.1	0.7	195.4
N4C2W1_C	213	**234.5**	1.6	199.8	241.7	1.8	194.2	239.7	6.8	191.4	241.3	2.0	201.5	238.1	1.9	198.3

**Table 8:** Normal distribution *z*-test.

Instance	Versus GA_1_	Versus GA_2_	Versus DGA_1_	Versus DGA_2_
br17	^ +^(3.16)	^ +^(3.16)	∗(0.00)	∗(0.00)
ftv33	^ +^(5.97)	^ +^(14.88)	^ +^(6.76)	^ +^(5.74)
ftv35	^ +^(7.67)	^ +^(6.35)	^ +^(7.68)	^ +^(8.79)
ftv38	^ +^(7.48)	^ +^(5.40)	^ +^(7.86)	^ +^(9.03)
p43	^ +^(8.07)	^ +^(10.26)	^ +^(8.85)	^ +^(7.60)
ftv44	^ +^(5.67)	^ +^(15.48)	^ +^(6.27)	^ +^(7.61)
ftv47	^ +^(6.10)	^ +^(5.93)	^ +^(6.67)	^ +^(7.84)
ry48p	^ +^(6.46)	^ +^(7.45)	^ +^(6.45)	^ +^(8.61)
ft53	^ +^(11.70)	^ +^(19.65)	^ +^(12.45)	^ +^(13.53)
ftv55	^ +^(6.77)	^ +^(8.18)	^ +^(10.57)	^ +^(12.19)
ftv64	^ +^(7.12)	^ +^(26.02)	^ +^(8.77)	^ +^(11.13)
ftv70	^ +^(6.10)	^ +^(21.97)	^ +^(9.89)	^ +^(13.83)
ft70	^ +^(2.28)	^ +^(25.62)	^ +^(4.88)	^ +^(6.65)
kro124p	^ +^(9.80)	^ +^(14.48)	^ +^(12.17)	^ +^(8.89)
ftv170	^ +^(3.64)	^ +^(3.16)	^ +^(4.69)	^ +^(4.79)
rbg323	^ +^(7.97)	^ +^(7.56)	^ +^(6.98)	^ +^(3.85)
rbg358	^ +^(35.52)	^ +^(35.54)	^ +^(12.54)	^ +^(11.73)
rbg403	^ +^(18.11)	^ +^(13.14)	^ +^(11.56)	^ +^(11.43)
rbg443	^ +^(10.76)	^ +^(10.05)	^ +^(10.96)	^ +^(10.84)

C101	^ +^(3.80)	^ +^(3.46)	^ +^(6.54)	^ +^(2.62)
C201	^ +^(9.75)	^ +^(13.49)	^ +^(13.93)	^ +^(3.08)
R101	^ +^(7.46)	^ +^(5.79)	^ +^(7.96)	∗(0.89)
R201	^ +^(21.16)	^ +^(8.18)	^ +^(11.39)	^ +^(5.29)
RC101	^ +^(10.32)	^ +^(7.96)	^ +^(5.74)	^ +^(5.83)
RC201	^ +^(10.16)	^ +^(6.26)	^ +^(14.93)	∗(1.45)
En23k3	^ +^(9.16)	^ +^(8.94)	^ +^(9.18)	∗(1.32)
En30k4	^ +^(18.97)	^ +^(17.84)	^ +^(9.77)	^ +^(7.44)
En33k4	^ +^(8.98)	^ +^(6.48)	^ +^(7.53)	^ +^(6.29)
En51k5	^ +^(9.70)	^ +^(10.49)	^ +^(14.61)	^ +^(2.38)
En76k8	^ +^(10.57)	^ +^(8.96)	^ +^(7.06)	^ +^(5.44)
En101k14	^ +^(13.24)	^ +^(7.93)	^ +^(18.47)	^ +^(10.24)

8-queen	∗(0.00)	∗(0.00)	∗(0.00)	∗(0.00)
20-queen	^ +^(13.00)	∗(0.00)	^ +^(7.91)	^ +^(6.08)
50-queen	^ +^(19.71)	^ +^(17.16)	^ +^(28.74)	^ +^(16.99)
75-queen	^ +^(31.37)	^ +^(15.71)	^ +^(33.25)	^ +^(22.16)
100-queen	^ +^(37.42)	^ +^(23.04)	^ +^(34.32)	^ +^(22.37)
125-queen	^ +^(31.57)	^ +^(31.97)	^ +^(39.71)	^ +^(36.39)
150-queen	^ +^(29.69)	^ +^(30.04)	^ +^(33.13)	^ +^(46.36)
200-queen	^ +^(33.09)	^ +^(33.66)	^ +^(36.14)	^ +^(34.96)
225-queen	^ +^(33.05)	^ +^(58.64)	^ +^(55.42)	^ +^(34.05)
250-queen	^ +^(61.21)	^ +^(26.80)	^ +^(43.06)	^ +^(51.66)
275-queen	^ +^(24.21)	^ +^(37.31)	^ +^(30.14)	^ +^(37.75)
300-queen	^ +^(60.37)	^ +^(40.71)	^ +^(45.51)	^ +^(37.27)
325-queen	^ +^(60.93)	^ +^(39.41)	^ +^(72.50)	^ +^(58.97)
350-queen	^ +^(66.00)	^ +^(52.06)	^ +^(59.30)	^ +^(43.39)
400-queen	^ +^(34.01)	^ +^(31.60)	^ +^(41.51)	^ +^(41.40)

N1C1W1_A	^ +^(6.32)	^ +^(11.06)	^ +^(10.11)	^ +^(8.85)
N1C1W1_B	^ +^(14.23)	^ +^(6.32)	^ +^(6.32)	^ +^(6.32)
N1C2W1_A	^ +^(9.39)	^ +^(9.39)	^ +^(9.89)	^ +^(12.72)
N1C2W1_B	^ +^(17.61)	^ +^(14.14)	^ +^(9.89)	^ +^(8.22)
N2C1W1_A	^ +^(19.79)	^ +^(13.19)	^ +^(17.91)	^ +^(9.96)
N2C1W1_B	^ +^(9.71)	^ +^(10.72)	^ +^(12.73)	^ +^(11.33)
N2C2W1_A	^ +^(7.00)	^ +^(17.61)	^ +^(18.00)	^ +^(14.00)
N2C2W1_B	^ +^(8.09)	^ +^(12.49)	^ +^(16.19)	^ +^(14.57)
N3C2W2_A	^ +^(28.59)	^ +^(15.64)	^ +^(20.95)	^ +^(14.48)
N3C2W2_B	^ +^(40.08)	^ +^(19.89)	^ +^(19.29)	^ +^(16.90)
N3C3W1_A	^ +^(32.34)	^ +^(9.55)	^ +^(14.74)	^ +^(8.04)
N3C3W1_B	^ +^(20.57)	^ +^(7.98)	^ +^(14.14)	^ +^(8.48)
N4C1W1_A	^ +^(24.07)	^ +^(12.23)	^ +^(21.45)	^ +^(17.64)
N4C2W1_A	^ +^(24.98)	^ +^(2.48)	^ +^(27.58)	^ +^(15.01)
N4C2W1_B	^ +^(17.97)	∗(1.27)	^ +^(26.22)	^ +^(21.00)
N4C2W1_C	^ +^(18.90)	^ +^(4.70)	^ +^(16.79)	^ +^(9.16)

^+^GB is significantly better. ^−^It is worse. ∗The difference is not significant (at 95% confidence level).

**Table 9:** Results of Friedman's test (smaller is better). The last column depicts the *X*_*r*_^2^ value.

Problem	GB	GA_1_	GA_2_	DGA_1_	DGA_2_	*X*_*r*_^2^
ATSP	**1.01**	3.16	4.52	3.17	3.01	42.04
VRPB	**1.00**	4.33	3.25	4.00	2.25	28.00
NQP	**1.04**	4.21	2.28	4.21	2.64	15.49
BPP	**1.00**	3.96	3.03	4.06	2.81	34.00
